# Genetic profiling of autoinflammatory disorders in patients with periodic fever: a prospective study

**DOI:** 10.1186/s12969-015-0006-z

**Published:** 2015-04-10

**Authors:** Carlo De Pieri, Josef Vuch, Eleonora De Martino, Anna M Bianco, Luca Ronfani, Emmanouil Athanasakis, Barbara Bortot, Sergio Crovella, Andrea Taddio, Giovanni M Severini, Alberto Tommasini

**Affiliations:** Institute for Maternal and Child Health IRCCS Burlo Garofolo, Trieste, Italy; University of Trieste, Trieste, Italy

**Keywords:** Auto-inflammatory diseases, Periodic fever syndromes, Genetics

## Abstract

**Background:**

Periodic fever syndromes (PFS) are an emerging group of autoinflammatory disorders. Clinical overlap exists and multiple genetic analyses may be needed to assist diagnosis. We evaluated the diagnostic value of a 5-gene sequencing panel (5GP) in patients with undiagnosed PFS.

**Methods:**

Simultaneous double strand Sanger sequencing of MEFV, MVK, TNFRSF1A, NLRP3, NLRP12 genes was performed in 42 patients with unexplained PFS. Clinical features were correlated with genetic results.

**Results:**

None of 42 patients analyzed displayed a causative genotype. However, single or multiple genetic variants of uncertain significance were detected in 24 subjects. Only in 5 subjects a definite diagnosis was made by taking into account both genetic and clinical data (2 TRAPS syndrome; 2 FMF; 1 FCAS). Statistical analysis showed that patients carrying genetic variants in one or more of the five selected genes displayed a significantly lower response to glucocorticoids compared with subjects who had completely negative genetic results.

**Conclusions:**

The sequencing of multiple genes is of little help in the diagnostics of PFS and can often lead to results of uncertain interpretation, thus the clinically driven sequencing of single genes should remain the recommended approach. However, the presence of single or multiple genetic variants of uncertain significance, even if not allowing any specific diagnosis, correlated with a poorer response to glucocorticoids, possibly indicating a multifactorial subgroup of PFS with differential response to pharmacological treatment.

**Electronic supplementary material:**

The online version of this article (doi:10.1186/s12969-015-0006-z) contains supplementary material, which is available to authorized users.

## Background

Periodic fever syndromes (PFS) are an emerging group of auto-inflammatory disorders [1]. Several well defined disorders with monogenic inheritance (hereditary periodic fevers HPF) have been described, including Familial Mediterranean Fever (FMF, OMIM #134610), Hyper-IgD/Mevalonate Kinase Deficiency syndrome (MKD, OMIM #610377 and #260920), tumor necrosis factor alpha associated periodic fever (TRAPS, OMIM #142680), familial cold-induced auto-inflammatory syndrome (FCAS, OMIM #120100) and a phenotype associated with NLRP12 mutations (FCAS2, OMIM #611762) [2]. These disorders arise from an improper control of inflammation, due to mutations in inflammasome-related proteins (such as pyrin in FMF, cryopyrin and NLRP12 in FCAS), or to defect in other proteins indirectly leading to abnormal inflammasome activation (such as mevalonate kinase in MKD and TNF-receptor in TRAPS) [3]. In typical cases, clinical criteria driving the attention toward a single candidate gene can assist the diagnostic process [4,5]. However, patients may often present incomplete phenotypes or overlap signs consistent with multiple pathological conditions. In addition, periodic fever adenitis pharyngitis aphthae syndrome (PFAPA), which is a quite common disorder without a definite genetic background, can also share some clinical features with the monogenic forms of HPS [6]. In most cases, prevalent involvement of pharynx, dramatic response to low dose glucocortioids and complete healing after tonsillectomy are distinctive signs confirming the diagnosis of PFAPA. In some atypical cases, however, poor response to steroids and/or persisting recurrence of fever after tonsillectomy will question the diagnosis. Moreover, patients with periodic fever displaying clinical pictures not supportive of any specific syndrome are being increasingly encountered in clinical practice. Accordingly, a scoring system with a decisional algorithm has been proposed for such ambiguous cases to facilitate the selection of patients for specific genetic analysis [5,7]. However, even after a careful selection, many patients remain without a genetic diagnosis and often undergo sequence analysis for multiple PFS genes [8].

For these reasons, given also the availability of improved techniques for genetic analysis with lowered costs, we developed an assay for complete sequencing of the five genes mostly involved in PFS (5-gene profile, 5GP),as listed in recent recommendations [9]. We applied this assay to patients with periodic fever testing negative to a previous genetic analysis as well as to patients with atypical PFAPA syndrome (defined on the basis of very early onset and/or poor response to glucocorticoids or tonsillectomy) and patients with other indefinite forms of periodic fever.

## Methods

### Patients

Patients (all with European genetic background) consecutively referred to the IRCCS Burlo Garofolo (Trieste, Italy) unit of pediatric rheumatology from March 2012 to April 2013 for periodic fever were enrolled in the study. Three groups were identified based on the clinical history: patients with a definite clinical picture of a monogenic PFS (Group 1); patients with a clinical picture of PFAPA syndrome (Group 2); other undefined periodic fevers (Group 3).

Group 1 included patients with specific suspect of monogenic PFS based on the following criteria: i. Tel Hashomer or Livneh criteria for Familial Mediterranena Fever; ii. Recurrent fever, lymphadenopathy and diarrhea with high levels of IgD or IgA or increased urinary mevalonate for Mevalonate Kinase Deficiency; iii. episodes of duration longer than 1 week and presence of myalgia or periorbital edema for TRAPS; iv. fever and urticaria following exposure to cold for Familial cold associated periodic syndrome (FCAS) [9].

Diagnosis of PFAPA syndrome (Group 2) was based on the presence of periodic fever and tonsillo-pharyngitis associated to cervical adenitis and/or aphthae, with onset after 6 months, not associated with infection from group-A beta hemolytic streptococcus.

Group 3 included all patients with unexplained periodic fever presenting clinical pictures that were supportive neither for PFAPA nor for any specific hereditary periodic fever syndrome.

Genetic investigation by means of the 5-genes panel (5GP) was proposed to: i. all patients in group 1 analyzed for a single candidate gene without detection of causative mutations (Group 1b); all patients with PFAPA who displayed onset before 6 months or poor response to glucocorticoids and/or to tonsillectomy (atypical-PFAPA, Group 2b); iii. All patients in group 3. For the purpose of this study, PFAPA was not considered atypical in cases with onset of the disease after 5 years of age.

For each enrolled subject a standardized questionnaire was filled, based on direct interviews and medical records (Additional file 1). The following information were collected: episode duration and clinical features, including symptoms, laboratory and imaging investigation results, response to treatments (steroids, colchicine).

Two patients who received alternative diagnosis during the follow-up, were excluded from the analysis.

### Genetic analysis

5GP assay. Briefly, genomic DNA was extracted from peripheral blood leukocytes and all coding regions and exon-intron boundaries regions of MEFV, MVK, TNFRSF1A, NLRP3, NLRP12 genes were amplified in a single plate using 59 primer pairs with a touchdown polymerase chain reaction as reported elsewhere [10]. Bi-direction sequencing has been performed using BigDye v3.1 Terminator Cycle Sequencing kit (Life Technologies, Foster City, CA) supplied with Betaine, (final concentration: 1 M), to ameliorate the amplification of GC-rich regions [11], and products were sequenced on ABI 3500Dx Genetic Analyzer (Life Technologies, Foster City, CA, USA). Sequences of both strands were imported to the SeqScape v2.7 software (Life Technologies, Foster City, CA, USA), aligned and compared with the published genes sequence reported in the database National Center for Biotechnology Information Database (NCBI). Detailed sequencing protocol is provided on line in supplementary materials (Additional file 1: 2a).

The interpretation of the genetic findings was performed according to the recommendations of an international consensus of experts [12] for the most common variants in MVK, MEFV, TNFRSF1A and NLRP3. For rarer or novel variants in all genes, included NLRP12, we referred to data from literature and to on line tools (see Additional file 1: 2b).

### Statistical analysis

For statistical purposes, genetic results were considered “*uncertain*” in cases with: detection of single sequence variants of uncertain significance; detection of multiple sequence variants of uncertain significance as suggested in the guidelines for the genetic diagnosis of hereditary recurrent fevers of the European Molecular Genetics Quality Network [12]; single heterozygous mutations for autosomal recessive disorders, considered that in rare cases a dominant inheritance and digenic inheritance has been recently reported both for Familial Mediterranean Fever and for Mevalonate Kinase Deficiency, although with atypical phenotypes [13-15]. On the other hand, results were considered completely “*negative*” if no missense variation or only common single-nucleotide polymorphisms with no proven functional consequences were detected. By analyzing the clinical differences between cases with “uncertain” compared with “negative” results we aimed to evaluate the possible role of low penetrance or multiple variants in the pathogenesis of multifactorial PFS.

Categorical data are presented as number and percentage, continuous data as median and interquartile range (IQR). To evaluate the association between the genetic outcome dichotomized as *uncertain* and *negative* (see above) and possible associated variables (sex, age, duration of episodes, presence, frequency and intensity of abdominal pain, presence and frequency of diarrhea and thoracic pain, presence and intensity of aphthosis, presence of fever, myalgia, rash, conjunctivitis, periorbital oedema, arthralgia or synovitis, peritonitis, pleurisy, pericarditis, nephritis, proteinuria, splenomegaly, response to corticosteroids, response to colchicine, cold induced crisis, urticaria, urethritis, the chi square test, or the Fisher exact test when appropriate, were used for categorical variables and the non parametric Mann Whitney test for continuous variables. The use of non parametric tests is justified by the not normal distribution of data evaluated both visually and with the Kolmogorov-Smirnov test. To identify the variables strongly and independently associated with the negative outcome a multivariate logistic regression analysis (forward stepwise method) was carried out. A p value of less than 0.05 indicates a statistically significant difference. The data were analyzed using SPSS for Windows 21.0.

## Results

### Groups of enrolled patients and selection of cases for 5GP genetic analysis

**Figure 1:**
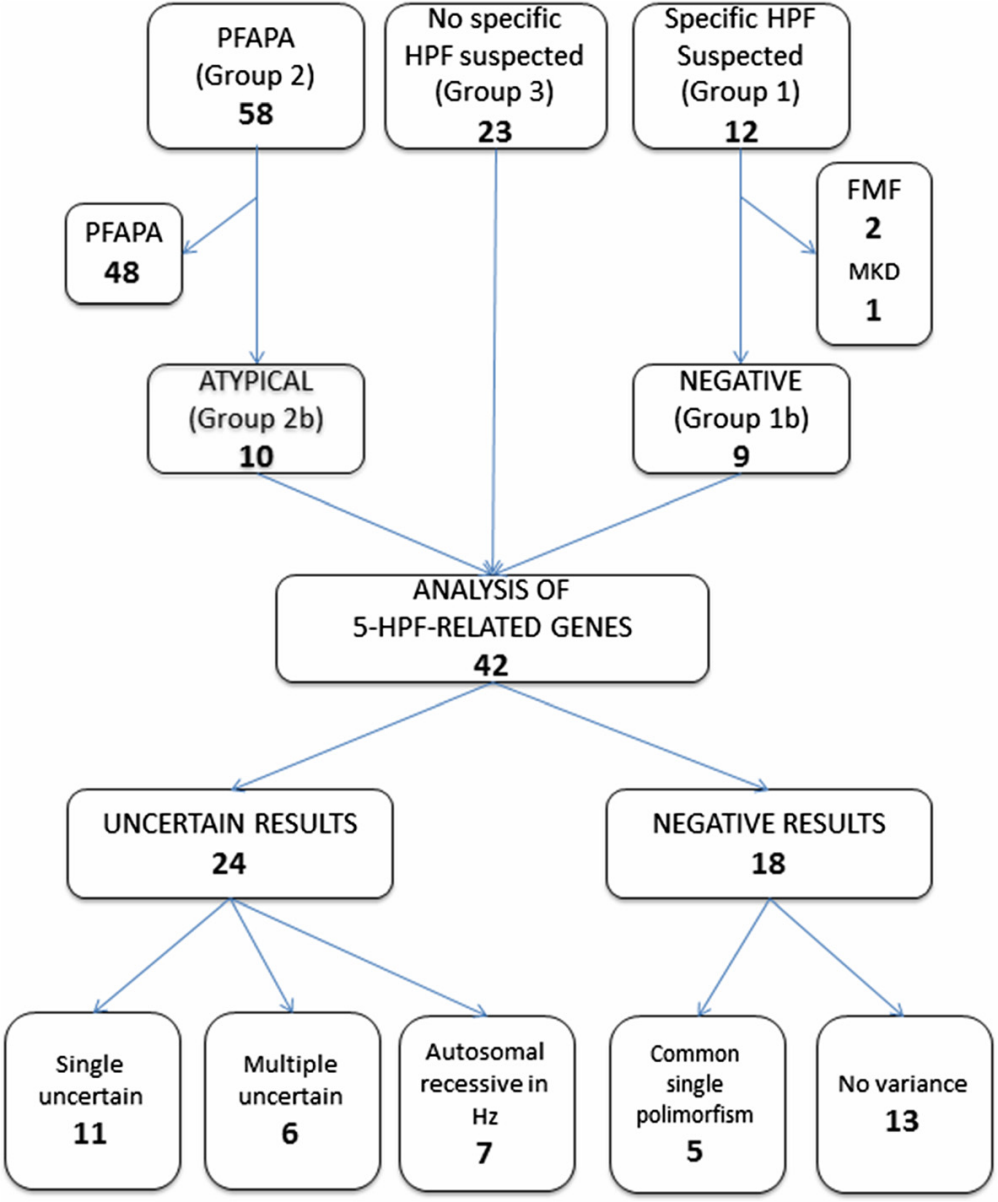
**Patients who underwent the analysis of the 5 HPF- related genes.**

A total of 93 patients were enrolled in the study. Among these subjects, 12 were suspected of a specific monogenic PFS (Group 1), 58 received the diagnosis of PFAPA syndrome (Group 2), and 23 had a clinical picture not specific of any definite PFS (Group 3, Figure 1).

The 12 subjects from Group 1 underwent single gene analysis. MVK was investigated in 7 subjects, MEFV in 4, TNFRSF1A in 1. One patients was found homozygous for the c.1129G > A (V377I) mutation in MVK and diagnosed as MKD. Two subjects carrying M680I (rs28940580 NM_000243.2:c.2040G > C)/V726A (rs28940579 NM_000243.2:c.2177 T > C) and M694V(rs61752717 NM_000243.2:c.2080A > G/R761H (rs104895097 NM_000243.2:c.2282G > A) mutations in MEFV were diagnosed as FMF. These three subjects, carrying high penetrance genotypes sufficient for a definite diagnosis,were excluded from the following study. The remaining 9 patients (2 male, 7 female, mean age 12,3 years) entered the study (Group 1b).

Among 58 subjects referred to the rheumatology unit for periodic fever with the clinical picture of PFAPA (Group 2), 10 have been found to display atypical features (2 male, 8 female, mean age 6,9 years). In particular, 5 showed poor response to steroids, 3 relapsed after tonsillectomy and 2 had disease onset before 6 months of age (Group 2b). Moreover, 2 subjects classified as atypical-PFAPA also had the disease onset after 5 years of life.

**Figure 2:**
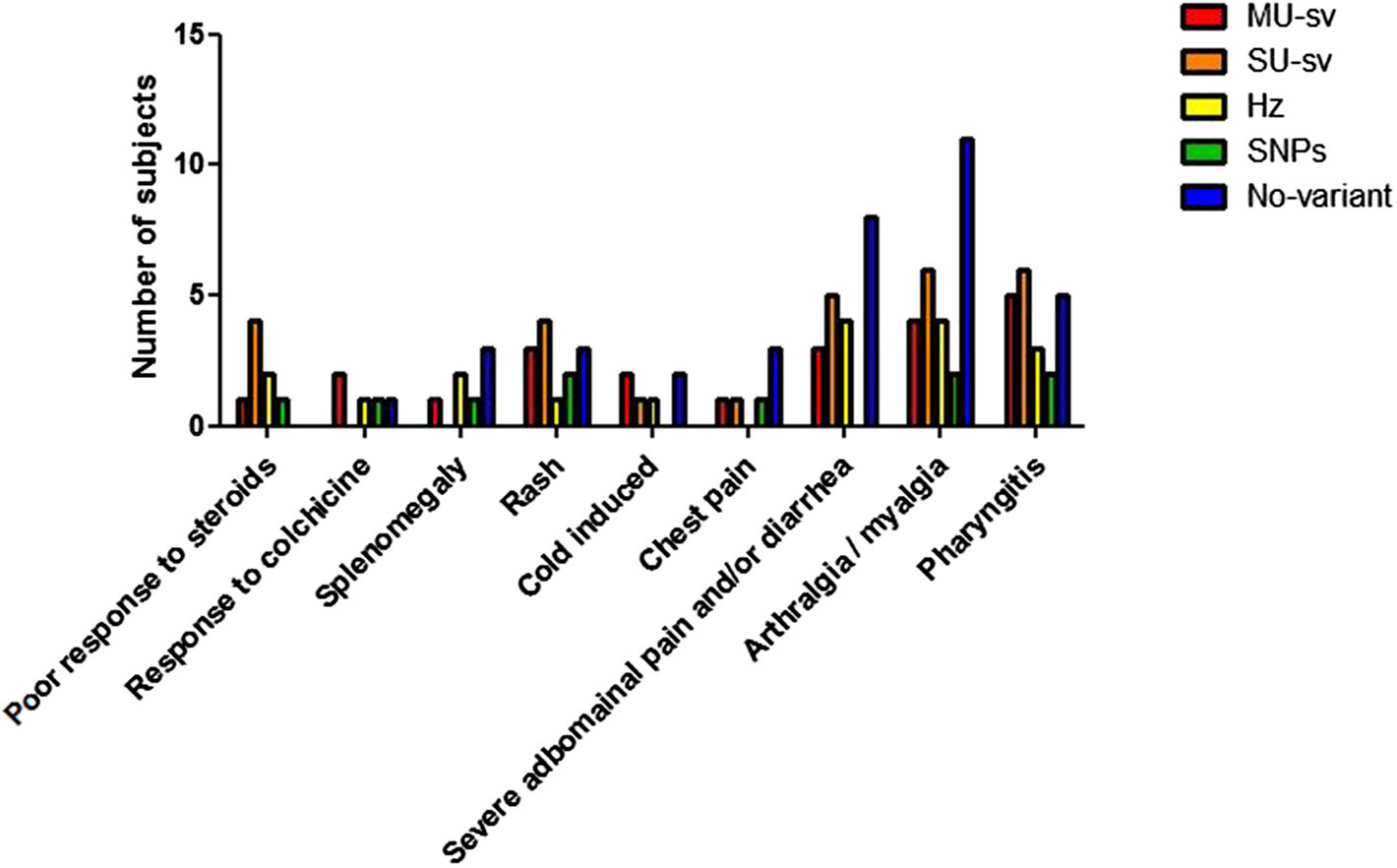
**Descriptive differences between all the genotype subset.** Legend: MU: multiple uncertain, SU: single uncertain, Hz: heterozygous, SNP: single nucleotide polymorphism.

Group 3 was composed by 23 subjects showing undefined clinical picture with periodic fever and multisystemic complaints (15 male, 8 female, mean age 12,5 years). The main clinical features are summarized in Table 1 and Figure 2.

**Table 1:** **Main clinical features in patients with uncertain genetic results**

**Symptoms**	**Uncertain genetic results (n = 24)**	**Univariate OR (95% CI)**	**p value**
**Response to corticosteroids, n (%)** ^**#**^			
Yes	7/19 (36.8)	0.2 (0.04-0.9)	0.03
No	10/13 (76.9)		
**Myalgia, n (%)**			
Yes	9/21 (42.9)	0.3 (0.1-1.1)	0.06
No	15/21 (71.4)		
**Abdominal pain and/or diarrhea, n (%)**			
Yes	19/29 (65.5)	3.0 (0.8-11.8)	0.1
No	5/13 (38.5)		
**Arthralgia, n (%)**			
Yes	11/22 (50.0)	0.5 (0.2-1.9)	0.3
No	13/20 (65.0)		
**Tonsillitis, n (%)**			
Yes	14/22 (63.6)	1.8 (0.5-6.0)	0.4
No	10/20 (50.0)		
**Toracic pain, n (%)**			
Yes	2/6 (33.3)	0.3 (0.1-2.0)	0.4^f^
No	22/36 (61.1)		
**Rash, n (%)**			
Yes	7/14 (50.0)	0.6 (0.2-2.4)	0.5
No	17/28 (60.7)		
**Cold-induced crisis, n (%)**			
Yes	3/4 (75.0)	2.4 (0.2-25.5)	0.6^f^
No	21/38 (55.3)		
Response to colchicine, n (%)*			
Yes	3/5 (60.0)	1.1 (0.1-11.6)	1.0^f^
No	4/7 (57.1)		
**Splenomegaly, n (%)**			
Yes	3/6 (50.0)	0.7 (0.1-4.0)	1.0^f^
No	21/36 (58.3)		

^#^Available for 32 subjects receiving corticosteroids.

^f^Fisher exact test.

*Available for 12 subjects receiving colchicine.

### Genetic results

The 5GP assay was performed in 42 subjects: 9 from Group 1b, 10 from Group 2b and 23 from Group 3 (Figure 1).

**Figure 3:**
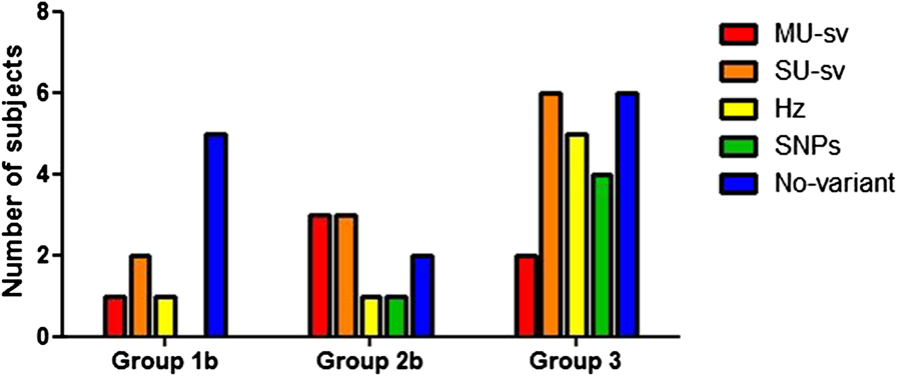
**Clinical differences according to genetic results.** Legend: MU: multiple uncertain, SU: single uncertain, Hz: heterozygous, SNP: single nucleotide polymorphism.

A clearly pathogenic genotype was found in none of 42 subjects. Twenty-four individuals had genetic results of uncertain significance. In particular 11 subjects carried single variants of uncertain significance in one of the five genes analyzed, 6 subjects had variants of uncertain significance in more than one gene analyzed and 7 were heterozygous for clearly pathogenic mutations associated with recessive disorders (Table 2, Figure 3).

**Table 2:** **Genotypes and interpretation of patients with uncertain genetic results**

	**GENE/cDNA accession**	**rs ID/mutation**	**MAF (1000 g)**	**Interpretation**
1	NLRP12/NM_144687.2	rs141245482/c.910C > T H304Y	< 0.01	Single mutations of uncertain significance
2	NLRP3/NM_001079821.2	rs142651552/c.2024G > A Q705K		
3	NLRP12/NM_144687.2	rs34971363/c.1206C > G; F402L	0.03	
4	NLRP12/NM_144687.2	rs34971363/c.1206C > G; F402L	0.03	
5	NLRP12/NM_144687.2	rs34971363/c.1206C > G; F402L	0.03	
6	NLRP3/NM_001079821.2	rs142651552/c.2024G > A Q705K		
7	TNFRS1A/NM_001065.3	rs4149584/c.362G > A; R121Q	0.01	
8	NLRP12/NM_144687.2	rs34971363/c.1206C > G; F402L	0.03	
9	NLRP12/NM_144687.2	rs34971363/c.1206C > G; F402L	0.03	
10	NLRP12/NM_144687.2	rs34971363F/c.1206C > G; F402L	0.03	
11	TNFRS1A/NM_001065.3	rs4149584/c.362G > A; R121Q	0.01	
1	MEFV/NM_000243.2	rs224222/c.602G > A; R202Q	0.17	Multiple mutations of uncertain significance
	MEFV/NM_000243.2	rs224222/c.602G > A; R202Q*	0.17	
2	MVK/NM_000431.2	rs7957619/c. 155G > A S52N	*0.09*	
	MVK/NM_000431.2	rs7957619/c. 155G > A S52N	*0.09*	
3	NLRP12/NM_144687.2	rs34971363/c.1206C > G; F402L	0.03	
	NLRP3/NM_001079821.2	rs142651552/c.2024G > A; Q705K	NA	
	MEFV/NM_000243.2	rs224222/c.602G > A; R202Q	0.17	
	MVK/NM_000431.2	rs7957619/c. 155G > A S52N	0.09	
4	NLRP12/NM_144687.2	rs34971363/c.1206C > G; F402L	0.03	
	MEFV/NM_000243.2	rs224222/c.602G > A R202Q	0.17	
*5*	NLRP3/NM_001079821.2	rs142651552/c.2024G > A Q705K	0.17	
	MEFV/NM_000243.2	rs224222/c.602G > A R202Q		
6	NLRP3/NM_001079821.2	rs142651552/c.2024G > A Q705K	0.02	
	MEFV/NM_000243.2	rs11466023/c.1105C > T; P369S	0.02	
	MEFV/NM_000243.2	rs11466024/c.1223G > A; R408Q		
1	MEFV/NM_000243.2	rs61732874/c.2230G > T; A744S	<0.01	Heterozygous mutations associated with autosomal recessive disorders
2	MEFV/NM_000243.2	rs3743930/c.442G > C E148Q	0.08	
3	MEFV/NM_000243.2	rs11466045/c.1772T > C; I591T	0.01	
4	MEFV/NM_000243.2	rs224222/c.602G > A R202Q	0.17	
	MEFV/NM_000243.2	rs61752717/c.2080A > G M694V	0.09	
	MVK/NM_000431.2	rs7957619/c. 155G > A S52N		
5	MEFV/NM_000243.2	rs3743930/c.442G > C E148Q	0.08	
	MEFV/NM_000243.2	rs224222/c.602G > A R202Q	0.17	
6	NLRP12/NM_144687.2	rs34436714 c.116G > T; *G39V*	0.25	
	MVK/NM_000431.2	rs28934897 c.1129G > A V377I		
*7*	MEFV/NM_000243.2	rs224222/c.602G > A R202Q	0.17	
	MVK/NM_000431.2	rs28934897 c.1129G > A V377I		

*Although hetherozygous R202Q variant in MEFV is considered a common polymorphism not to be reported, the same variant in homozygosis has been associated to cases of Familial Mediterranean Fever in Greece [32].

There was no significant difference in the frequency of genetic variants in the three groups even if heterozygous clearly pathogenic mutations, as defined in the European Molecular Genetics Quality Network giuidelines [12], tended to be more common among Group 3. Tables 1 and 2 (Additional file 1: 3) show the results of the univariate analysis carried out to evaluate the association between the genetic result and clinical signs and symptoms. The response to corticosteroids was statistically significantly lower in children with uncertain genetic results (36.8% vs 76.9%, OR 0.2, 95% CI 0.04-0.9, p = 0.03). Multivariate logistic regression analysis confirmed this result, showing that only this variable was associated to the uncertain genetic results.

Eighteen subjects were wild type (13) or only showed common non-pathogenic polymprphism (5).

### Alternative diagnosis during the follow up

After one year follow-up, different diagnosis emerged in two cases that consequently were excluded from the study. A 17 years old boy referred to the unit of pediatric rheumatology for periodic fever and systemic symptoms. Febrile episodes during follow-up were no more associated with increased acute phase reactants. The boy was successively referred to our neuropsychiatric unit where a Münchhausen syndrome was eventually diagnosed.

A 10 years old girl who resulted completely negative at the genetic analysis was successively referred to the hematology-oncology unit of our Institute, where a diagnosis of Castleman disease was done. The girl entered the study due a multisystemic picture (group 3) characterized by onset at 9 years of age, recurrent and long during fever (mean duration 15 days), frequent abdominal pain, myalgia, splenomegaly and tonsillitis. The patient showed a moderate response to glucocorticoids.

## Discussion

Periodic fever syndromes encompass a wide spectrum of disorders, ranging from common multifactorial ones, such as PFAPA syndrome, to more rare monogenic disorders [16-18]. Thanks to increased awareness of these diseases among physicians and to improved molecular diagnostics, PFS are increasingly detected in patients cared by various specialists for undefined inflammatory disorders. Indeed, PFS explain a significant proportion of recurrent fever of unknown origin in recent series [19].

Genetic testing for a number of PFS recently provided a valuable tool to corroborate uncertain clinical diagnosis, resulting in increased detection and registration of cases into international registries [20].

Since clinical overlap of symptoms with variable intensity and localization is described in PFAPA and in monogenic PFS, decisional algorithms have been developed to assist the selection of cases for genetic analysis [7]. Furthermore, given the identification of a large number of genetic variants in PFS genes, a consensus document was released, with recommendation for the screening and interpretation of genetic results for PFS [12].

However, a recent retrospective study identified a large proportion of patients categorized as PFS based on their clinical features, in whom the genetic workup was either negative or inconclusive [8].

We obtained similar results in our prospective study, showing that a large proportion of cases with recurrent fever not associated to conclusive results from genetic analysis may be encountered in pediatric practice. During the period of the study, a conclusive clinical and genetic diagnosis of PFS was obtained only in three subjects who underwent genetic studies for a single candidate gene. On the contrary, a clearly pathogenic genotype was not found in any of the subjects enrolled in the 5GP study, suggesting that a polygenic or multifactorial pathogenesis may be involved in most of them. Of note, given the selection criteria of our study, the common multifactorial PFAPA syndrome can account only for a minor proportion of these cases. In fact, patients with recurrent fever and pharyngitis were included only if displaying atypical features such as poor response to steroids, relapse after tonsillectomy or onset in the first 6 months of life and represented just a quarter of our series. Indeed, most patients presented clinical pictures not supportive of any specific PFS, while some patients had FMF, MKD or TRAPS-like phenotype. However, a variety of uncertain or inconclusive results were found in many subjects, ranging from single variant of uncertain significance, multiple variants and single heterozygous mutations associated with recessive disorders. We questioned if subjects with uncertain results actually represent a distinct group compared with subjects with completely negative genetic results as concern clinical phenotype and response to treatments. Statistical analysis showed a poorer response to glucocorticoids in subjects with any combination of uncertain variants compared with negative cases, while no significant difference was found as regards other symptoms. A possible explanation is that a disease continuum exists between typical monogenic and multifactorial PFS, and that some of the cases with incomplete or uncertain genotypes may represent intermediate forms between the two ends that can display reduced response to glucocorticoids.

In fact, FMF-like PFS has been reported in subjects with a single heterozygous mutation in MEFV and in rare cases a dominant pattern of inheritance has been described [13,15,21]. Actually, the recent observation of FMF-like PFS in subjects with double heterozygous mutations in MEFV and NLRP3 raises the hypothesis that some cases could be due to digenic or polygenic inheritance [14,22]. Increasing use of genetic approaches with simultaneous analysis of multiple PFS genes will clarify this issue in the next future.

However, the clinical phenotype associated with heterozygous MEFV mutation is usually mild or atypical and a definite diagnosis of FMF can be done only if clinical criteria of the disease are satisfied [15]. In our series, the heterozygous mutation c.442G > C in *MEFV* leading to the aminoacid substitution E148Q was found in a young man with recurrent bouts of fever and abdominal pain that responded to colchicine and in a young girl with atypical-PFAPA, who showed spontaneous improvement during follow-up. Moreover, the complex allele c.1105C > T- c.1223G > A (P369S-R408Q) was found in a girl with recurrent fever and Behcet-like complaints, but with poor response to colchicine.

The most common variants of uncertain significance were the c.1206C > G variation (F402L) in NLRP12, and the c.2024G > A (Q705K) variant in NLRP3. Recent data suggest that these variants can affect the function of the respective proteins, however these findings are controversial [23-25].

Furthermore these variants have been found with quite high frequency in general European population, making very difficult their correlation with clinical diagnosis (www.1000genomes.org/). Indeed, the frequency of these variants in our series tends to be higher than in general population, although the difference is not statistically significant (data not shown). However, our study was not intended to establish if specific variants are associated or not with PFS, rather to investigate if they can identify subgroups of PFS with distinctive features.

Some patients display multiple variants in different genes. Even if we cannot exclude that these association is due to chance, it is possible that they contribute to the disease in a polygenic or multifactorial way. Digenic pathogenesis of PSF has been recently hypothesized in cases with double heterozygous mutations in different genes [13-15,26].

Another possibility is that some cases are due to mutations in other yet unidentified genes or to somatic mosaicisms not detectable with conventional genetic sequencing. This possibility has been well documented for mutations in NLRP3 that even when expressed in low percentages of cells can lead to inflammatory amplification and severe phenotypes [27-31].

Whatever the significance of the detected genotypes, our data confirm that non-specific PFS can be encountered in pediatric practice. In most cases, the phenotype is milder compared to most monogenic PFS, but more serious than the common PFAPA syndrome. In fact, during one year of follow up, spontaneous resolution occurred only in 2 cases, one of them tried to simulate febrile illnesses also after resolution. Indeed, we carefully considered the possibility of simulation to explain persistence of symptoms in some cases, yet only in one case normal values of erythrocyte sedimentation rate during crisis allowed disclosing the factious nature of the fever.

Dealing with patients affected by PFS without definite clinical and genetic diagnosis represents a novel challenge, in particular as concern therapeutic options, follow-up evaluation and prognosis. Some patients are empirically treated based on the clinical and or genetic analogy with the well defined PFS. For example, colchicine was administered to 13 patients in our series, with good response in five, regardless of the underlying genotype identified.

Tonsillectomy is a possible option for subjects with atypical PFAPA syndrome with poor response to glucocorticoids and high frequency of crisis, however response to surgery may be diminished in these cases, as suggested by the presence of three patients relapsed after tonsillectomy in our series.

## Conclusions

In our experience, a target gene sequencing approach including five PFS-associated genes showed very limited diagnostic advantages in the diagnostics of PFS, and in fact it often led to results of difficult interpretation. Thus, the analysis of single genes should remain the preferred approach to the diagnostics of PFS. In addition, we showed that a large proportion of subjects with periodic fevers without a definite genetic background, based on our current knowledge, can be commonly encountered in the clinical practice of a tertiary care department. Longer time of follow-up and, possibly novel genetic evaluations will clarify the clinical significance of these group of “undifferentiated” PFS.

## Additional file

Additional file 1:
**1) Questionnaire; 2a) Genetic analysis protocol; 2b) Mutation data handling; 3) Supplementary tables.**

## Abbreviations

PFS: Periodic fever syndromes
5GP: Five gene panel
TRAPS: Tumor necrosis factor alpha associated periodic syndrome
FMF: Familial Mediterranean fever
FCAS: Familial cold associated syndrome
HPF: Hereditary periodic fevers
MKD: Mevalonate kinase deficiency
FCAS2: Familial cold associated syndrome type 2
PFAPA: Periodic fever adenitis pharyngitis aphthae syndrome
IQR: Interquartile range
