# Supplementary material for: Genetic profiling of autoinflammatory disorders in patients with periodic fever: a prospective study
Source: Pediatr Rheumatol Online J. 2015 Apr 10;13:11. doi: 10.1186/s12969-015-0006-z (PMC4393620; doi:10.1186/s12969-015-0006-z)
Supplement: Additional file 1: — 1) Questionnaire; 2a) Genetic analysis protocol; 2b) Mutation data handling; 3) Supplementary tables. [file 12969_2015_6_MOESM1_ESM.docx]

**Additional file**

1) Questionnaire

2a) Genetic analysis protocol

2b) Mutation data handling

3) Supplementary tables

**1) Questionnaire**

**Date …………………… Physician**

**Personal data**

First Name

Last Name

Sex

Date of birth

Place of birth

Mother’s place of birth

Father’s place of birth

**Clinical features**

Age at the onset (month)

Average duration of the crisis (day)

Fever (0 = no 1 = yes)

Abdominal pain frequency (0 = never, 1 = sometimes, 2 = often, 3 = every day)

Abdominal pain intensity (0= no, 1 = mild, 2 = moderate, 3 = severe)

Diarrhea (0 = never, 1 = sometimes, 2 = often, 3 = every day)

Myalgia (0 = no 1 = yes)

Rash (0 = no 1 = sì)

Conjunctivitis (0 = no 1 = yes)

Periorbital edema (0 = no 1 = sì)

Toracic pain frequency (0-3)

Arthralgia or synovitis (0 = no 1 = yes)

Aphthosis (0 assente, 1 minor, 2 major)

Peritonitis (0 = no 1 = yes)

Pleurisy (0 = no 1 =yes)

Cold- induced crisis (0 = no 1 = yes)

Tonsillitis (0 = no 1 = yes)

Splenomegaly (0 = no 1 = yes)

Mesenteric adenopathy (0 = no 1 = yes)

Urinary mevalonic acid (0 no data, 1 = negative 2 = positive)

Increased IgA / IgD value (0 = no data, 1 = negative 2 = positive)

Responds to steroids (0 = no data, 1 = negative 2 = positive)

Responds to colchicine (0 = no data, 1 = negative 2 = positive)

Family history(0 no; 1 yes) (explain)

**2a)Genetic analysis protocol**

**Isolation of DNA**

Genomic DNA was extracted from peripheral blood leukocytes by the QIAamp DNA Blood Mini kit (Qiagen, Hilden, Germany). The quantity and the quality of the DNA was assessed using Nanodrop ND-1000 spectrophotometer (Nanodrop, Wilmington, USA).

**Primer design**

To adapt the sequencing strategy of the genes *MEFV*, *MVK*, *TNFRSF1A*, *NLRP3,* *NLRP12* to the analysis of single samples, a rapid protocol was developed as previously described by Bortot *et al*. (2011)^1^, allowing PCR amplification and sequencing reactions to be carried out under identical conditions for all 59 reactions in a 96-well reaction plate for each sample. All coding regions and exon-intron boundaries regions were amplified using 59 primer pairs designed using the web tool Primer3Plus (Untergasser *et al*., 2007)^2^. The primer design was performed to a melting temperature of 58-66°C and 40-70 percentage of GC. Primer sequences are available on request.

**Touchdown polymerase chain reaction and sequencing**

The initial sequencing strategy involved an automated set-up, employing a Biomek NX MC liquid handling platform (Beckman Coulter, CA, USA), as reported by Bortot *et al*. All PCR primers (59 primer pairs) were distributed in two 96-well plate, 2 μM of each primer, ones for the Forward primers and the other for the Reverse. For the automated protocol, PCR was carried out in 10 μl total reaction volume. Each reaction contained 25 ng of genomic DNA and Kapa 2G Fast Hot Start ReadyMix 2X (KapaBiosystems, Cape Town, South Africa). PCR amplification was performed using a common annealing temperature in a touch down thermocycler protocol shared in two cycle step as reported elsewhere (Bortot *et al*). All thermal cycle steps, PCR amplifications, ExoSAP purifications and sequencing reactions, were performed on a GeneAmp PCR System 9700 Thermocycler (Life Technologies, Foster City, CA).

Sequencing reaction of all 59 PCR products has been performed using BigDye v3.1 Terminator Cycle Sequencing mixture (Life Technologies, Foster City, CA) supplied with Betaina, (final concentration: 1M), to ameliorate the amplification of GC-rich regions (Henke *et al*., 1997)^3^, for a total volume of 10 μl. After purification with BigDye XTerminator Purification Kit (Life Technologies, Foster City, CA), sequence products were sequenced bidirectionally on ABI 3500Dx Genetic Analyzer (Life Technologies, Foster City, CA, USA). All identified mutations were confirmed by repeated sequencing of both DNA strands of the affected exon on a second PCR product.

**2b) Mutation data handling**

Sequencing data were imported to the SeqScape v2.7 software (Life Technologies, Foster City, CA, USA) and compared to the published genes sequence database National Center for Biotechnology Information Database (NCBI). To classify all variants identified were consulted the public databases: NCBI dbSNP build138 (http://www.ncbi.nlm.nih.gov/SNP/), 1000 Genomes Project (http://www.1000genomes.org/), and NHLBI-ESP (<http://evs.gs.washington.edu/EVS/>). To test the pathogenicity of all novel missense variants, SIFT (http://sift.jcvi.org/), PolyPhen-2 (http://genetics.bwh.harvard.edu/pph2/), MutationTaster (http://www.mutationtaster.org/), and MutationAssessor (http://mutationassessor.org/), web tools for the prediction of potential impact of an amino acid substitution on the structure and function of a human protein, were used. To detect alterations in exon–intron boundary regions and splicing motifs due to nucleotide changes, were consulted the bioinformatic tools for splice site prediction HSF (http://www.umd.be/HSF) and NNSplice (http://www.fruitfly.org/seq_tools/splice.html). All the unknown variants were tested among 50 ethnically-matched control samples.

**3) Supplementary tables**

| **Symptoms** | **Uncertain genetic results (n=24)** | **Univariate OR**  **(95% CI)** | **p value** |
| --- | --- | --- | --- |
| **Sex, n (%)** |  |  |  |
| Female | 14/23 (60.9) | 1.4 (0.4-4.8) | 0.6 |
| Male | 10/19 (52.6) |  |  |
| **Aphthosis, n (%)** |  |  |  |
| Yes | 8 /18 (44.4) | 0.4 (0.1-1.4) | 0.2 |
| No | 16/24 (66.7) |  |  |
| **Fever, n (%)** |  |  |  |
| Yes | 24/42 (57.1) | - | - |
| **Conjunctivitis, n (%)** |  |  |  |
| Yes | 1/3 (33.3) | 0.3 (0.03-4.2) | 0.6 ^f^ |
| No | 23/39 (59.0) |  |  |
| **Periorbital oedema, n (%)** |  |  |  |
| Yes | 4/7 (57.1) | 1.0 (0.2-5.2) | 1.0 ^f^ |
| No | 20/35 (57.1) |  |  |
| **Peritonitis, n (%)** |  |  |  |
| Yes | 1/1 (100.0) | - | 1.0 ^f^ |
| No | 23/41 (56.1) |  |  |
| **Pleurisy, n (%)** |  |  |  |
| Yes | 1/2 (50.0) | 0.7 (0.04-12.7) | 1.0 ^f^ |
| No | 23/40 (57.5) |  |  |
| **Pericarditis, n (%)** |  |  |  |
| Yes | 0/3 (0.0) | - | 0.08 |
| No | 23/38 |  |  |
| **Nephritis, n (%)** |  |  |  |
| No | 24/42 (57.1) | - | - |
| **Proteinuria, n (%)** |  |  |  |
| Yes | 0/1 (0.0) | - | 0.4 ^f^ |
| No | 24/41 (58.5) |  |  |
| **Urticaria, n (%)** |  |  |  |
| Yes | 4/4 (100.0) | - | 0.1 ^f^ |
| No | 20/38 (52.6) |  |  |
| **Urethritis, n (%)** |  |  |  |
| Yes | 1/1 (100.0) | - | 1.0 ^f^ |
| No | 23/41 (56.1) |  |  |
| **Intensity of abdominal pain, n (%) *** |  |  |  |
| Mild | 8/10 (80.0) | 0.3 (0.1-2.2) | 0.4 ^f^ |
| Moderate/Severe | 8/14 (57.1) |  |  |
| **Diarrhoea, n (%)** |  |  |  |
| Yes | 8/11 (72.7) | 2.5 (0.6-11.2) | 0.3 ^f^ |
| No | 16/31 (51.6) |  |  |
| **Frequency of diarrhoea, n (%)°** |  |  |  |
| Sometimes | 4/6 (66.7) | 0.5 (0.03-8.0) | 1.0 ^f^ |
| Often/ every day | 4/5 (80.0) |  |  |
| **Intensity of thoracic pain, n (%)°°** |  |  |  |
| Mild | 2/5 (40.0) | - | 1.0 ^f^ |
| Moderate/Severe | 0/1 (0.0) |  |  |
| **Aphthosis, n (%)°°°** |  |  |  |
| Minor | 5/14 (35.7) | 0.2 (0.02-2.3) | 0.3 ^f^ |
| Major | 3/4 (75.0) |  |  |

^f^ Fisher exact test

* available for 24 subjects with abdominal pain

° available for 11 subjects with diarrhoea

°° available for 6 subjects with thoracic pain

°°° available for 18 subjects with aphthosis

eTable 1: Additional clinical variables evaluated at univariate analysis

|  | **Uncertain genetic results (n=24)** | **Negative genetic results (n=18)** | **p*** |
| --- | --- | --- | --- |
| Age at onset of disease in months, median (IQR) | 16.0 (6.0-108.0) | 24.0 (5.0-96.0) | 0.8 |
| Duration of episodes in days, median (IQR) | 4.3 (2.9-5.9) | 4.5 (2.9-7.3) | 0.8 |

* Mann-Whitney test

eTable 2: Age at onset and duration of episodes comparison between uncertain and negative genetic results

**Bibliography**

1 Bortot B, Athanasakis E, Brun F, Rizzotti D, Mestroni L, Sinagra G, Severini GM.

High-throughput genotyping robot-assisted method for mutation detection in patients with hypertrophic cardiomyopathy.

Diagn Mol Pathol. 2011 Sep;20(3):175-9. doi: 10.1097/PDM.0b013e31820b34fb.

2 [Untergasser A](http://www.ncbi.nlm.nih.gov/pubmed?term=Untergasser%20A%5BAuthor%5D&cauthor=true&cauthor_uid=17485472), [Nijveen H](http://www.ncbi.nlm.nih.gov/pubmed?term=Nijveen%20H%5BAuthor%5D&cauthor=true&cauthor_uid=17485472), [Rao X](http://www.ncbi.nlm.nih.gov/pubmed?term=Rao%20X%5BAuthor%5D&cauthor=true&cauthor_uid=17485472), [Bisseling T](http://www.ncbi.nlm.nih.gov/pubmed?term=Bisseling%20T%5BAuthor%5D&cauthor=true&cauthor_uid=17485472), [Geurts R](http://www.ncbi.nlm.nih.gov/pubmed?term=Geurts%20R%5BAuthor%5D&cauthor=true&cauthor_uid=17485472), [Leunissen JA](http://www.ncbi.nlm.nih.gov/pubmed?term=Leunissen%20JA%5BAuthor%5D&cauthor=true&cauthor_uid=17485472).

Primer3Plus, an enhanced web interface to Primer3.

[Nucleic Acids Res.](http://www.ncbi.nlm.nih.gov/pubmed/?term=Primer3Plus%2C+an+enhanced+web+interface+to+Primer3) 2007 Jul;35(Web Server issue):W71-4. Epub 2007 May 7.

3 [Henke W](https://posta.um.fvg.it/owa/redir.aspx?C=3TPExHZSTE6ZCdmrgqTo8BwSQfanftEIS9ogfuqI--KXzIFrGosgSW6fsZR8qxWp3kgWC6hszqk.&URL=http%3a%2f%2fwww.ncbi.nlm.nih.gov%2fpubmed%3fterm%3dHenke%2520W%255BAuthor%255D%26cauthor%3dtrue%26cauthor_uid%3d9380524), [Herdel K](https://posta.um.fvg.it/owa/redir.aspx?C=3TPExHZSTE6ZCdmrgqTo8BwSQfanftEIS9ogfuqI--KXzIFrGosgSW6fsZR8qxWp3kgWC6hszqk.&URL=http%3a%2f%2fwww.ncbi.nlm.nih.gov%2fpubmed%3fterm%3dHerdel%2520K%255BAuthor%255D%26cauthor%3dtrue%26cauthor_uid%3d9380524), [Jung K](https://posta.um.fvg.it/owa/redir.aspx?C=3TPExHZSTE6ZCdmrgqTo8BwSQfanftEIS9ogfuqI--KXzIFrGosgSW6fsZR8qxWp3kgWC6hszqk.&URL=http%3a%2f%2fwww.ncbi.nlm.nih.gov%2fpubmed%3fterm%3dJung%2520K%255BAuthor%255D%26cauthor%3dtrue%26cauthor_uid%3d9380524), [Schnorr D](https://posta.um.fvg.it/owa/redir.aspx?C=3TPExHZSTE6ZCdmrgqTo8BwSQfanftEIS9ogfuqI--KXzIFrGosgSW6fsZR8qxWp3kgWC6hszqk.&URL=http%3a%2f%2fwww.ncbi.nlm.nih.gov%2fpubmed%3fterm%3dSchnorr%2520D%255BAuthor%255D%26cauthor%3dtrue%26cauthor_uid%3d9380524), [Loening SA](https://posta.um.fvg.it/owa/redir.aspx?C=3TPExHZSTE6ZCdmrgqTo8BwSQfanftEIS9ogfuqI--KXzIFrGosgSW6fsZR8qxWp3kgWC6hszqk.&URL=http%3a%2f%2fwww.ncbi.nlm.nih.gov%2fpubmed%3fterm%3dLoening%2520SA%255BAuthor%255D%26cauthor%3dtrue%26cauthor_uid%3d9380524). Betaine improves the PCR amplification of GC-rich DNA sequences. [Nucleic Acids Res.](https://posta.um.fvg.it/owa/redir.aspx?C=3TPExHZSTE6ZCdmrgqTo8BwSQfanftEIS9ogfuqI--KXzIFrGosgSW6fsZR8qxWp3kgWC6hszqk.&URL=http%3a%2f%2fwww.ncbi.nlm.nih.gov%2fpubmed%2f%3fterm%3dBetaine%2bImproves%2bthe%2bPCR%2bAmplification%2bof%2bGC-Rich%2bDNA%2bSequences%23) 1997 Oct 1;25(19):3957-8.
